# The evolving genetic landscape of neuromuscular fetal akinesias

**DOI:** 10.1177/22143602251339357

**Published:** 2025-05-13

**Authors:** Göknur Haliloğlu, Gianina Ravenscroft

**Affiliations:** 1 Division of Pediatric Neurology, Department of Pediatrics, Hacettepe University Faculty of Medicine, Ankara, Turkey; 2 Centre for Medical Research, Faculty of Health and Medical Sciences, The University of Western Australia, Perth, Western Australia, Australia; 3 Rare Disease Genetics and Functional Genomics, Harry Perkins Institute of Medical Research, Perth, Western Australia, Australia

**Keywords:** fetal akinesia, arthrogryposis, arthrogryposis multiplex congenita, multiple pterygia syndrome, congenital contractures, neuromuscular, neurogenetics

## Abstract

Fetal akinesia is a broad term used to describe absent (or reduced, fetal hypokinesia) fetal movements, and it can be detected as early as the first trimester. Depending on the developmental age of onset, anything that interferes or limits the normal *in utero* movement results in a range of deformations affecting multiple organs and organ systems. Arthrogryposis, also termed arthrogryposis multiplex congenita (AMC), is a definitive terminology for multiple congenital contractures, with two major subgroups; amyoplasia and distal arthrogryposis (DA). The spectrum includes fetal akinesia deformation sequence (FADS), lethal congenital contracture syndrome (LCCS), and multiple pterygium syndrome (MPS). Variants in more than >400 genes are known to cause AMC, and it is increasingly recognized that variants in genes encoding critical components (including ventral horn cell, peripheral nerve, neuromuscular junction, skeletal muscle) of the extended motor unit underlie ∼40% of presentations. With unbiased screening approaches, including sequencing of comprehensive disease gene panels, exomes and genomes, novel genes and phenotypic expansions associated with known human disease genes have been uncovered in the setting of fetal akinesia. Autosomal-recessive titinopathy is the most frequent genetic cause of AMC. Accurate genetic diagnosis is critical to genetic counseling and informing family planning. Around 50% remain undiagnosed following comprehensive prenatal, diagnostic or research screening. Comprehensive phenotyping and periodic reanalysis with appropriate genomic tools are valuable strategies when faced with initial inconclusive results. There are likely many novel causative genes still to identify, which will inform our understanding of the molecular pathways underlying early human development and *in utero* movement.

## Introduction

Fetal akinesia is a broad term used to describe absent (or reduced, fetal hypokinesia) fetal movements. Embryonic and fetal movements are defined as an integral part of typical development, and there is a close association between activity and development of central and peripheral structures.^1,2^ Evolution of generalized and isolated movements are elegantly discussed in a recent review, highlighting the importance of using dynamic imaging modalities as a complementary tool to understand ontogeny of fetal motor functions and maturational deviations within a critical period of development.^2^ Understanding the sequence of these movements in relation to duration of lack of *in utero* movements, help to tie physical examination findings and deformations in multiple organ and organ systems.^3–6^ Pregnancy can be complicated by polyhydramnios or oligohydramnios. Fetal akinesia deformation sequence (FADS), which was originally described as ‘Pena Shokeir’ phenotype, and lethal congenital contracture syndrome (LCCS) represent the severe end of this spectrum.^3–6^

Arthrogryposis or the extended term, arthrogryposis multiplex congenita (AMC) is a definitive term, and is characterized by multiple joint contractures affecting at least two different unrelated body parts presenting at birth.^3–9^ The prevalence of AMC is estimated at ∼1:3000–5000 live births,^10,11^ with FADS representing a rare subset of these cases and a prevalence between 1:7000–25,000.^12^

Any part of the neurological and neuromuscular anatomical structures (central nervous system (CNS), spinal cord, ventral horn cell, peripheral nerve, neuromuscular junction, muscle) alongside connective tissue including tendons, joints and bone may be involved, and result in fetal akinesia and AMC. To date, there is no universally adapted fully comprehensive classification system, but separating extrinsic and intrinsic factors, and a multilayered approach based on clinical, etiologic, genetic and functional classifications is useful.^3–13^ Traditional approaches distinguish amyoplasia and distal arthrogryposis (DA), and a further classification based on the presence or absence of CNS involvement, syndromic forms, chromosomal abnormalities, neurogenic (including both structural and functional nerve abnormalities), and myogenic (including both structural and functional abnormalities of muscle) origin. Given the range of insults or defects, extensive workup is needed to identify the likely disease mechanism.^3,4,14,15^

Variants in more than >400 genes (e.g., panelapp.genomicsengland.co.uk/panels/258/) are known to cause AMC, and thus far, gene ontology analysis reveals more than 19 gene clusters.^16^ Insights into the mechanisms and understanding biological process, molecular function and cellular component/s behind each gene involved is challenging, since genes have often more than one function and have different tissue expression, thus may involve multiple pathways. Moreover, expansion of the phenotypes in previously identified genes pose a challenge. For example, a loss-of-function variant in *ARL6IP1*, which was previously associated with a rare, complicated form of hereditary spastic paraplegia (HSP), is now identified in neurogenic AMC, hypotonia, and CNS involvement.^17^ Different inheritance patterns of the same gene may result in expansion of the phenotypes. For example, a loss-of-function variant in *THOC2*, a gene which was previously associated with X-linked intellectual disability, is now identified in fetal arthrogryposis with muscle biopsy showing cytoplasmic bodies.^18^

It is increasingly recognized that variants in genes encoding critical components of the neuromuscular system underlie a substantial proportion of fetal akinesia presentations, reviewed previously.^19–21^ In a recent series of fetal akinesia investigated by whole exome sequencing (WES), Lacquieriere et al. identified primary involvement of skeletal muscle in 40%, followed by CNS involvement in 22% of AMC cases later manifesting with epilepsy^14^ and intellectual disability.^22^ Fetal akinesia in concert with CNS involvement is frequent and overlapping phenotypes include syndromic forms and/or monogenic disorders with CNS and peripheral nervous system (PNS) involvement such as alpha-dystroglycan related congenital muscular dystrophies (CMDs), channelopathies, and malformations of cortical development.^23^

Despite advances in molecular genetics and the increasing number of identified genes, an associated gene alteration has been found in only about half of the patients (47–65%).^24–27^ In the era of massively parallel sequencing, many new genetic causes of fetal akinesia have been delineated and characterized, and these new genotype-phenotype associations have led to increased diagnostic yield. For example, the diagnostic yield for fetal akinesia and arthrogryposis from screening with bespoke, targeted gene panels increased from 31% to 43% as the number of genes included on the panel increased, indicating that many of these novel genes represent a substantial proportion of diagnoses, in contrast to other neuromuscular disease groups where diagnostic yield remained the same over the same iterative panels.^28^ Nevertheless, it is important also to consider the contribution of chromosomal microarray evaluation and genes which are less amenable to screening via massively parallel sequencing.

**Figure 1.**
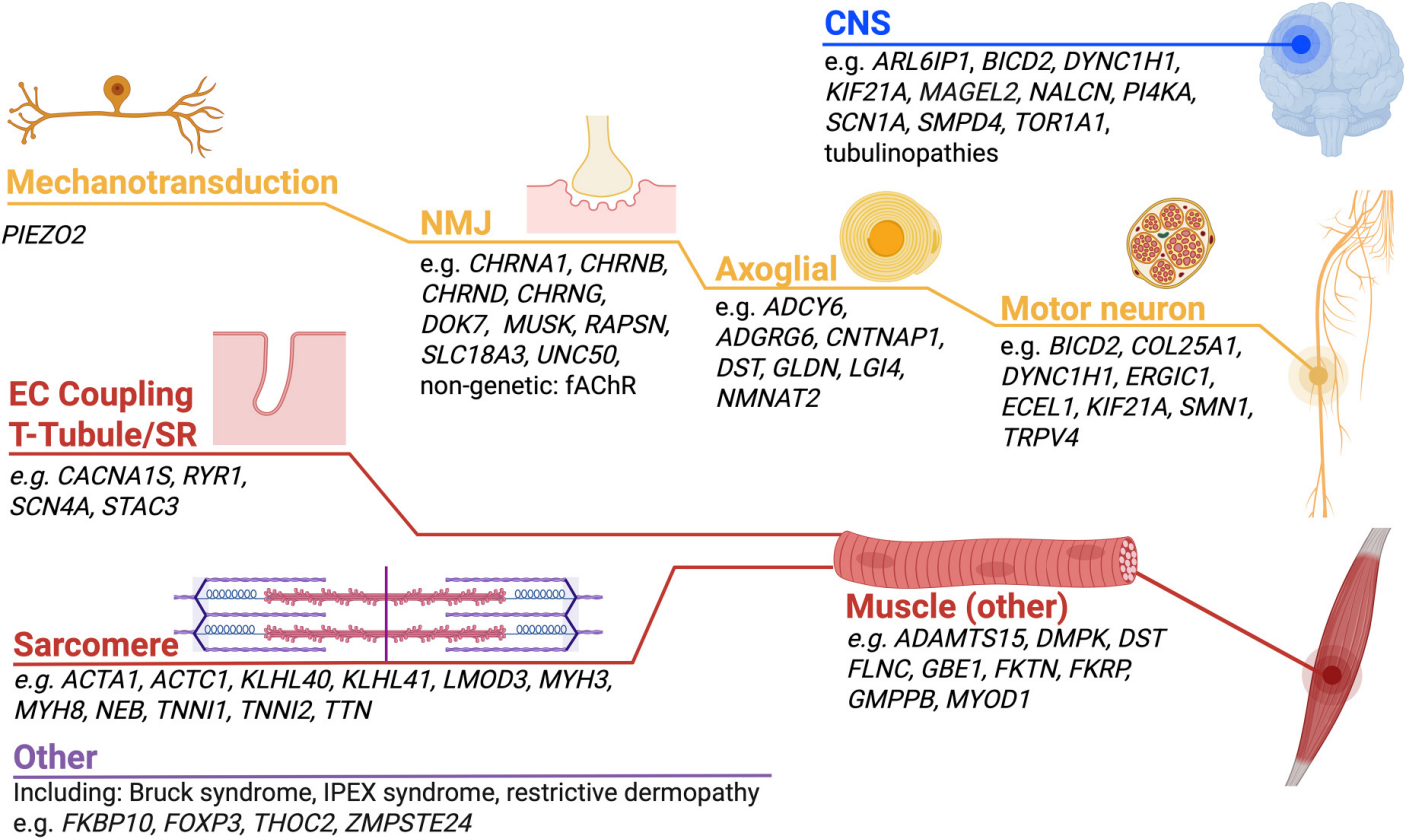
Schematic demonstrating the key pathways implicated in neuromuscular and neurogenetic fetal akinesias, with the most frequently causative genes and more recently described genes listed.

The main aim of this narrative review is twofold: to summarize the causative neuromuscular genes involved in fetal akinesia and associated disorders highlighting some examples based on topographical distribution presented in Figure 1, and to discuss current and emerging molecular diagnostic strategies.

### Common genetic neuromuscular disorders that may present as fetal akinesia and that may be missed with massively parallel sequencing approaches

Some forms of fetal akinesia can result from gene defects that may evade detection by short-read sequencing approaches, these include SMA type 0,^22,29,30^ and large repeat expansions in *DMPK* reviewed in Ho *et al*.^31^ Given the ever-increasing reliance of diagnostics via massively parallel sequencing, targeted gene panels, exomes or even genomes, it is important to consider the potential for these genes to underly fetal akinesia and to consider whether these genes are included in the sequencing and/or analysis of the ordered test. *SMN1* deletions are challenging to detect given the sequence homology with the *SMN2* pseudogene and the issues this creates for read alignment and calling of variants. Newer algorithms now exist and can detect *SMN1* variants from short-read sequencing data.^30^ *DMPK* expansions, when maternally inherited are particularly prone to further expansion and it is not uncommon for a minimally affected woman to have a severely affected child than can present as fetal akinesia.^4,32^ It is also conceivable that some molecularly unsolved fetal akinesias may result from repeat expansions at other loci, that are yet to be identified, and researchers should ensure short tandem repeats (STRs) calling, e.g., via ExpansionHunter *de novo*,^32^ is performed in unsolved cases.

*Central nervous system (CNS):* Intellectual disability and CNS involvement is found approximately in 25% of AMC cases reviewed by Dieterich et al.^23^ We would like to highlight a couple of examples that may serve as exemplars for overlapping CNS and PNS involvement.

### Prenatal onset muscular dystrophies, GMPPB as an exemplar

As with myotonic dystrophy, it is also recognized that variants in typical muscular dystrophy genes can also result in a fetal akinesia/hypokinesia presentation including for example *FKTN*, *FKRP* and *GMPPB**.*** Variants in *GMPPB*, encoding a cytoplasmic protein GDP-mannose pyrophosphorylase B, which catalyzes the formation of GDP-mannose required for the O-mannosylation of alpha-dystroglycan (DG), result in a spectrum varying from severe CMD with structural brain abnormalities to limb-girdle muscular dystrophy (LGMD), recurrent rhabdomyolysis without skeletal muscle weakness, asymptomatic hyper-CKemia and congenital myasthenic syndrome due to altered glycosylation of the acetylcholine receptor subunits and other synaptic proteins.^33–35^ Although, LGMD is the most common phenotype (60–70%) within this spectrum, prenatal presentation with abnormal intrauterine growth, decreased fetal movements, arthrogryposis, congenital clubfoot deformity, and scoliosis are recognized as part of early onset severe CMD presentations.^33,34^ In a recent series from Italy, two of 13 patients with variants in *GMPPB* presented with arthrogryposis, microcephaly, intellectual disability; one with congenital cataract, and the other with cerebellar polymicrogyria.^35^

### MAGEL2-associated arthrogryposis

Since 2013, truncating variants, inherited paternally, have been associated with Schaaf-Yang and Chitayat-Hall syndromes, both characterized by AMC.^36,37^ Other variable features include intellectual disability, dysmorphic features, hypopituitarism, growth hormone deficiency, intestinal pseudoobstruction, and obesity. *MAGEL2* is a key gene associated with Prader-Willi syndrome, a disorder of imprinting, and has been studied extensively; it encodes a ubiquitin ligase enhancer that is required for endosomal protein recycling and is highly expressed in the hypothalamus.^37^

### Channelopathies

The voltage-gated sodium channel isoforms expressed in either the muscle or the brain generate and conduct action potentials.^38^ Dysfunction of these channels leads to either a reduction (loss-of-function) or an increase (gain-of-function) in tissue excitability. Among these channelopathies, recessive *SCN4A* variants cause fetal akinesia, congenital myopathy and congenital myasthenia, with clinical severity depending on the degree of channel dysfunction.^39^ Primarily brain-expressed sodium channel genes, *SCN1A, SCN2A, SCN3A* and *SCN8A* have been associated with epilepsy and neurodevelopmental disorders.^38^ Recently, patients with antenatal diagnosis of AMC carrying *de novo SCN1A* variants, who either died *in utero* or developed neonatal-onset drug-resistant epileptic seizures were identified.^39–41^ This unique phenotype is further expanded by movement disorders and malformations of cortical development.^41–43^ Delineation of the characteristics of gain-of-function *SCN1A*-related disease, based on an international cohort of 35 patients, revealed three distinct presentations depending on age at onset and presence of arthrogryposis and movement disorders.^44^

NALCN is a conserved cation channel related to voltage-gated sodium and calcium channels. Loss-of-function variants in *NALCN* cause an autosomal recessive condition characterized by infantile hypotonia, psychomotor retardation and characteristic facial features.^45^ On the other hand, autosomal dominant *NALCN* variants are identified as a cause of congenital DA with pursed facial expression suggesting a hypercontracted phenotype and designated as congenital contractures of the limbs and face with hypotonia and developmental delay (CLIFAHDD).^46^ Modeling of the later variants indicate that both gain-of-function and loss-of-function changes may underlie the condition.^47^

### Peripheral nervous system (PNS): including motor neuron survival, specification, innervation and myelination

Neurogenic arthrogryposis can represent early-onset neuronopathies, reviewed recently,^48,49^ and most frequently arise due to autosomal dominant (usually *de novo*) variants in *BICD2*, *DYNC1H1* and *TRPV4*. These genes accounted for diagnoses in 21 of 505 families across two arthrogryposis cohorts.^22,28^

*ERGIC1 *encodes a putative transmembrane protein of the Endoplasmic Reticulum-Golgi Intermediate Compartment (ERGIC), that functions as a tubulovesicular membrane cluster in protein sorting and trafficking. Homozygous missense variants and a homozygous deletion encompassing the promoter and first exon of *ERGIC1* are identified as a cause of relatively mild non-syndromic arthrogryposis and neuropathic AMC.^27,50,51^ The pathogenic role of *ERGIC1* is further validated by mRNA studies revealing complete loss-of-function.^51^

Kinesin motor protein KIF21A, tubulin isotypes, or transcription factors are involved in motor neuron specification or motor nerve development. Falb *et al*., in a cohort of 23 cases with fetal akinesia, identified biallelic loss-of-function *KIF21A* variants in five affected fetuses of two unrelated families, and introduced *KIF21A* as a new factor underlying severe neurogenic FADS with arthrogryposis of multiple joints, pulmonary hypoplasia and facial dysmorphisms.^52^ This highlights the essential role of kinesins, in various cellular processes including cell-cycle dynamics and progression, ciliogenesis and cilia function, and organization of polar cells during organogenesis. This adds *KIF21A*, to the list of already known fetal akinesia and other disease-associated kinesin superfamily proteins such as KIF5C and KIF26B.^53,54^

Defects in intramuscular innervation are known to cause AMC due to variants in *ECEL1/DINE*.^55,56^ Natera-de Benito *et a*l., identified variants in *COL25A1* presenting with a recognizable phenotype characterized by AMC with or without an ocular congenital cranial dysinnervation disorder, belonging to this pathophysiological mechanism.^57^ *COL25A1* encodes a transmembrane-type collagen crucial for intramuscular motor innervation. Proper interaction between muscle-derived collagen XXV and its motor neuron-derived receptors protein tyrosine phosphatases are required for the navigation of motor axons into developing skeletal muscle.^58^ The first phenotype described due to recessively inherited pathogenic variants in *COL25A1* was ocular congenital cranial dysinnervation disorders (CCDDs) in four patients from two families.^59,60^ CCDSs, within the spectrum of axonal guidance disorders, result in absent or aberrant innervation of extraocular and/or cranial musculature. Phenotypic spectrum of *COL25A1*-related disorders is now expanded with a novel contractual phenotype, variable severity of arthrogryposis, ocular CCDD, without CNS involvement and relatively stable/slow progression and respiratory involvement in some of the patients, due to pathogenic homozygous and compound heterozygous variants.^57^ Collagen XXV is discussed to have a role in regulating the appropriate innervation not only of extraocular muscles, but also of bulbar, axial, and limb muscles in humans.

Defects in proteins critical to formation and function of the nodes of Ranvier can result in inherited nodopathies that present as fetal akinesia. *CNTNAP1* (OMIM 602346) encodes CASPR an essential component of the nodes of Ranvier that are critical to saltatory conduction of action potentials along myelinated axons. Biallelic loss-of-function variants in *CNTNAP1* result in fetal akinesia and non-syndromic AMC with reduced motor NCVs and structural defects in myelinated axons of peripheral nerves.^61,62^ CNS involvement in the form of cerebral and cerebellar atrophy is also reported.^63^ In 2016, the Melki group first described loss-of-function variants in *GLDN* (OMIM 608603) encoding gliomedin, another key protein of the nodes of Ranvier, as a cause of lethal arthrogryposis (LCCS 11).^64^ A cell-based assay that examines cell-surface localization of over-expressed tagged wild type and mutant gliomedin is a useful assay for accessing variants of uncertain significance, especially substitutions.^28,64,65^ *GLDN*-related phenotypes also include non-lethal presentations, with survival of patients into infancy, childhood and adolescence with intensive care and chronic respiratory and nutritional support.^66^

Loss-of-function variants in genes encoding signaling proteins that are critical to myelination of the peripheral nervous system underlie rare forms of AMC, including *LGI4*,^67^ *ADCY6*,^61^ and *ADGRG6* (formerly known as *GPR126*).^68,69^ Intrafamilial variability is also described in *LGI4*-related arthrogryposis.^70^ Examination of myelination of intramuscular nerve either by immunohistochemistry or electron microscopy can identify defective myelination of the PNS and may aid diagnosis.^68^

Biallelic loss-of-function variants in the dystonin gene (*DST*) were recently identified in a patient with arthrogryposis, in whom examination of sciatic nerve showed severe hypomyelination.^71^ Recessive variants in the neuronal isoform of *DST* have previously been associated with hereditary sensory and autonomic neuropathy (HSN6, MIM: 614653) with onset from the fetal to adult period. Jacob *et al*. described 17 arthrogryposis patients from 12 families with eight homozygous variants within the largest exon of *DST* encoding isoform DST-b. These patients presented with arthrogryposis, hypotonia and cardiac involvement, muscle biopsies were myopathic and it was found that DST-b is most abundant in striated muscle.^72^

Lukacs *et al*. identified bi-allelic hypomorphic variants in *NMNAT2* in stillborn siblings with FADS, amyoplasia and hydrops.^73^ Nicotinamide mononucleotide adenylyltransferases are required for synthesis of NAD ^+^ and are critical for cellular metabolism. Studies in model organisms show NMNAT2 is critical for survival of peripheral nerve axons.^65^ The phenotypic spectrum associated with biallelic *NMNAT2* variants extends to childhood-onset polyneuropathy with neurogenic pain.^73^

### Mechanotransduction

The first step in a biochemical cascade that translates mechanical inputs into cellular responses, ‘mechanotransduction’, is through the PIEZO2 cation channel.^74–76^ Gain-of-function variants in *PIEZO2* are known to cause autosomal dominant distal arthrogryposis syndromes; Marden-Walker syndrome (OMIM: 248700), DA type 3 (OMIM: 114300) and type 5 (OMIM: 108145).^77^ The so called ‘PIEZO2 deficiency’ due to biallelic loss-of-function variants in *PIEZO2* are identified as a cause of arthrogryposis syndrome with hypotonia, feeding difficulties, neonatal respiratory insufficiency, delayed motor development, facial dysmorphic features, impaired touch and proprioception, sensory ataxia, absence of deep tendon reflexes, progressive scoliosis, and abnormal skeletal phenotypes.^78–81^ Electrophysiological studies demonstrate decreased sensory nerve action potentials. In patients with congenital distal contractures, other than proprioceptive and sensory deficits, involvement of additional biological systems such as urinary dysfunction and diminished perception of urinary urgency, can serve as a clue to diagnose PIEZO2 deficiency syndrome.^76^

### Neuromuscular junction

Genes encoding subunits of the post-synaptic acetylcholine receptor (AChR) and proteins critical to assembly and clustering of the AChRs and neuromuscular junction (NMJ) formation and development also underlie presentations ranging from FADS to milder Escobar-variant MPS and DA. Biallelic variants in *CHRNA1*, *CHRNB1*,^28^ *CHRND*, *DOK7*, *MUSK*, *RAPSN* and *UNC50*^82^ represent ∼11–17% of genetically diagnosed fetal akinesia and arthrogryposis cases.^22,28^ These disorders are allelic with congenital myasthenic syndromes, recently reviewed by Ohno *et al*.^83^ Variants in the gene (*CHRNG*) encoding the fetal (gamma) subunit of the pentameric AChR cause lethal and non-lethal MPS and DA,^84^ and represents the most frequent causative NMJ gene in arthrogryposis cohorts (12/505 families).^22,28^ A nonsense variant in the gene (*SLC18A3*) encoding the presynaptic vesicular acetylcholine transporter (VAChT) causes FADS.^85^

In addition to inherited forms of NMJ-associated fetal akinesia, maternal antibodies to fetal AChR isoform (fAChR) can also result in fetal akinesia and arthrogryposis. Fetal AChR antibodies result in a milder myopathic form called ‘fetal acetylcholine receptor inactivation syndrome, (FARIS)’.^86^ The spectrum of phenotypes, termed ‘fetal acetylcholine receptor antibody-related disorders, (FARAD)’, resulting from antibodies to fAChR, diagnostic and treatment strategies were recently reviewed by Allen *et al*.^87^ In this multicenter cohort including 46 patients, contractures and AMC were identified in 31/46 (67.4%). Of interest, diagnosis of myasthenia gravis was not established in 50% of the mothers, and offspring death was due to severe arthrogryposis (7/46, 15.2%) in the antenatal period, or respiratory failure (4/46, 8.7%) during early infancy. Although there are knowledge gaps owing to rarity and under recognition of these conditions, AMC, transient myasthenia gravis of the newborn, and FARIS seem to represent a continuum, and recognition of FARAD is critically important to implement intensive immunomodulatory treatment during pregnancy in myasthenia gravis-affected mothers, and to consider oral salbutamol treatment option for symptomatic offspring survivors.

### Skeletal muscle – excitation-contraction coupling

Several key proteins are critical for relaying the signal received at the neuromuscular junction into Ca^2+^ release from the sarcoplasmic reticulum, with sarcoplasmic Ca^2+^ being the key regulator of skeletal muscle contraction. These included the voltage-gated sodium channel within the sarcolemma and T-tubules, encoded by the gene *SCN4A*, the voltage sensing ryanodine receptor (encoded by *RYR1*) within the T-tubules and the Ca^2+^ release channel (dihydropyridine receptor, DHPR) within the SR (encoded by *CACNA1S*). Among these channelopathies, recessive *SCN4A* variants cause fetal akinesia, congenital myopathy and congenital myasthenia, with clinical severity dependent on the degree of channel dysfunction.^88^ Biallelic variants in *RYR1*, frequently loss-of-function variants, are also associated with fetal akinesia,^89^ whilst dominantly inherited variants are associated with myopathy and susceptibility to malignant hyperthermia. Healthy parents that are carriers of *RYR1* variants that cause fetal akinesia may also be at risk of malignant hyperthermia, and this represents an important aspect of genetic counseling.^24,89,90^ Dominantly- and recessively inherited *CACNA1S* variants cause congenital myopathy, with fetal akinesia representing severe manifestation of *CACNA1S*-myopathy.^28,91,92^

STAC3 is another key protein involved in excitation-contraction coupling and is thought to play a critical role in the coupling of RYR1 and the DHPR at the muscle triad. A biallelic c.851G > C, p.Trp284Ser variant in *STAC3* was first identified to underly Native American myopathy.^93^ This and other *STAC3* variants have since been identified in other forms of myopathy and in fetal akinesia.^28,94^ A founder effect (attributed to a carrier rate of one in 56 of the p.Trp284Ser variant) has recently been described in southern Africa, with 38% of cases presenting with AMC.^94^ Biallelic, typically loss-of-function variants, in any one of these four proteins can result in a fetal akinesia presentation.^24,28,90–94^ Putative splice-altering variants can be investigated by RNA studies or minigene assays,^28^ however variants of unknown significance, in particular missense variants remain a challenge. Assays to investigate functional changes associated with variants in these genes, include electrophysiology and Ca^2+^ assays, however these are time-consuming and require a laboratory with these expertise.^88,89^

### Skeletal muscle – the sarcomere

Defects within the sarcomere can impair muscle contraction *in utero* and result in fetal akinesia and arthrogryposis, these include components of the thin and thick filaments and represent a substantial proportion of diagnoses (77/505 families), with autosomal-recessive titinopathy being the single-largest contributor (22/77 diagnoses).^22,28^

**Figure 2.**
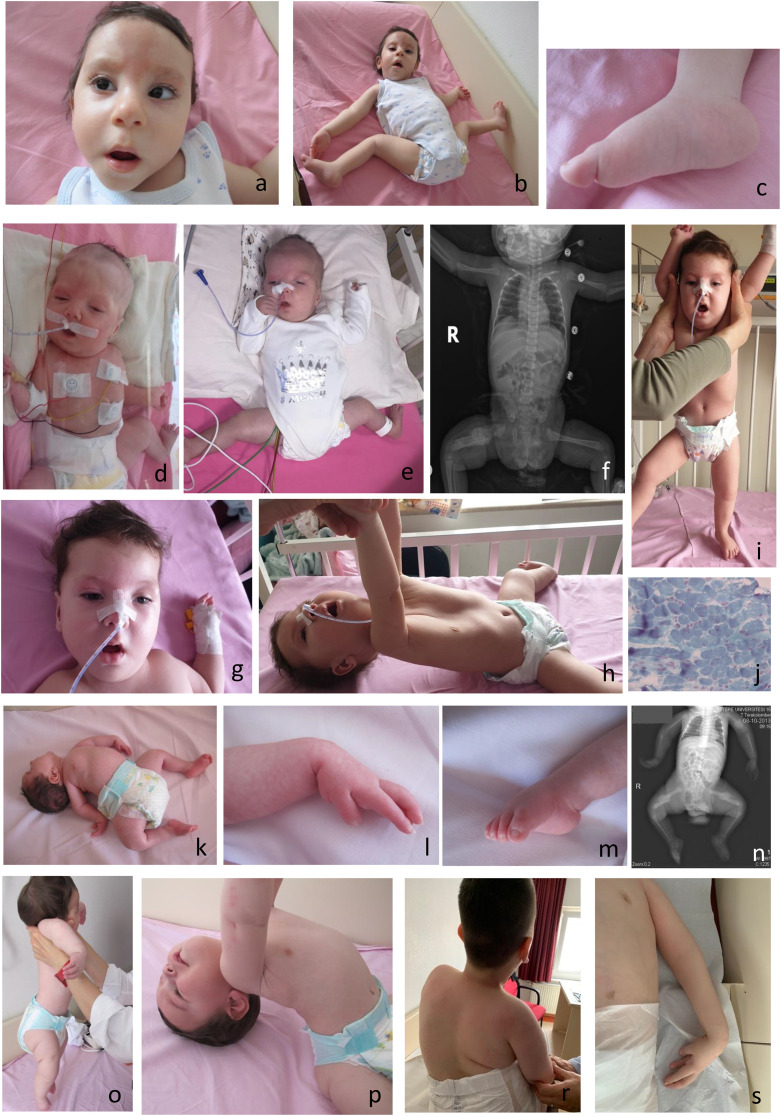
A 12-month-old infant with arthrogryposis at birth with a myopathic face, nevus glabellar and forehead nevus flammeus, esotropia, and typical posturing of the hands and feet (b, c) with a diagnosis of congenital titinopathy; an 11-month-old infant with *KLHL40*-related myopathy who presented with arthrogryposis multiplex congenita at birth (d), at the age of 3 months (e), right femur fracture at birth (f), and 11 months (g, h, i), respectively. Note myopathic face, mild ptosis, facial weakness with tented upper lip, clenched hands, external rotation and hyperabduction of the hip with lower extremity contractures (d, e), pectus excavatum deformity, hypotonia on traction and vertical suspension maneuvers, weakness (g, h, i), and muscle biopsy Gomori trichrome staining compatible with nemaline myopathy (j); a 11-year-old case with arthrogryposis multiplex congenita at birth with hyperextension of the neck (k), clenched hand (l), pes equinovarus deformity (m), multiple fractures at birth (n) with axial hypotonic weakness (o, p) and scoliosis, hand posturing and inverted nipple by the age of 11 years (r, s) with axonal neuropathy and neurogenic arthrogryposis.

It is increasingly recognized that biallelic truncating variants in *TTN* (*TTN*tv) (Figure 2) can present as fetal akinesia, AMC and recurrent miscarriage.^95–99^ Due to extensive alternative splicing of titin within skeletal and cardiac muscle, variants are often annotated in the inferred complete *TTN* meta-transcript, a theoretical isoform that includes all putative *TTN* exons. Many of causative variants underlying prenatal-onset disease occur in or alter exons (i.e., extended splice-variants) that are present only in the meta-transcript of *TTN*, i.e., they are not present in the canonical transcript.^96,97,99^ This includes a recurrent c.39974-11T > G splice variant.^96,97,99^ Contractures are most evident in upper extremities with elbows being most severely affected. The ‘meta-transcript only’ titinopathy has a recognizable contractural pattern characterized by ulnar abduction and extension of the wrist, persistent myopathy, and striking fibrofatty involvement of hamstrings and calves with relative sparing of the femoral adductors and anterior segment of thighs on muscle imaging.^100^ RNA studies have shown that these meta-transcript exons are more highly expressed in fetal skeletal muscle than adult skeletal muscle.^97,101^ In cases with a *TTN*tv in an exon expressed in cardiac *TTN*, cardiac involvement can also occur and may also manifest in the carrier parent and siblings.^99–101^

Causative variants in several genes are associated with nemaline myopathy.^102^ Autosomal recessive variants in *NEB*, the gene encoding nebulin - the giant sarcomeric protein of the thin filament, were initially associated with nemaline myopathy (NEM2),^103^ however many cases present with *in utero* onset of muscle weakness resulting in phenotypes of FADS, AMC, and lethal MPS, and/or cystic hygroma and cleft palate.^24,104–107^ Of note, variants in *KLHL40* (kelch-like family member 40) representing the severe and often lethal end of the nemaline myopathy spectrum (Figure 2) characterized by autosomal recessive FADS were first identified by our group.^108^ A recent systematic review, including 65 patients with *KLHL40* variants showed that, antenatal presentation (76%), fetal akinesia or hypokinesia (58%), contractures at birth (76%), and fractures (35%) dominated the clinical picture. Of the 65 patients described, 60% (n = 39) died during the first 4 years of life (intrauterine: n = 12; first year of life: n = 22; before 4 years of age: n = 5).^109^

Variants in genes encoding fetally expressed isoforms of sarcomeric proteins can result in fetal akinesia and arthrogryposis, including those encoding embryonic or perinatal myosin heavy chains, *MYH3* and *MYH8* respectively. Dominantly inherited or *de novo* missense variants in *MYH3* are well-established to underlie DA, in particular Freeman-Hall Syndrome and Sheldon-Hall Syndrome.^110^ More recently, dominant or recessive *MYH3* disease has been identified to cause a phenotype including contractures, pterygia and skeletal deformities, and to underlie spondylocarpotarsal synostosis syndrome characterized by intervertebral fusions and fusion of the carpal and tarsal bones.^111–113^ Biallelic missense variants in *MYH3*, both shown to alter splicing and predicted to result in loss of *MYH3* expression, and homozygous loss-of-function variants were also identified in cases with a lethal form of spondylocarpotarsal synostosis syndrome.^114^

*ACTC1* encodes cardiac alpha-actin of the sarcomere in the heart, this gene is also fetally expressed in skeletal muscle.^115^ Variants in *ACTC1* are typically associated with cardiomyopathies,^116^ however in 2023 Chong *et al*. identified a cohort of patients with DA and congenital heart defects arising from dominantly-inherited or *de novo* missense variants in *ACTC1*.^117^ Original clinical diagnoses included MPS, Sheldon-Hall or Freeman-Sheldon syndromes. All identified substitutions in *ACTC1* in Chong *et al*. have previously been observed in *ACTA1*, encoding skeletal muscle alpha-actin, a well-known congenital myopathy gene. *De novo* or somatic mosaic *ACTA1* variants are another well-described cause of fetal akinesia and arthrogryposis, with 5% of all *ACTA1* variants associated with ‘fetal abnormalities’ in a recent *ACTA1* review.^118^ Moreover, identification of a *de novo* pathogenic variant in *ACTA1* and prenatal brain abnormalities including enlarged subarachnoid space and delayed cortical development in FADS suggests a role for skeletal muscle alpha-actin in the CNS.^119^

Troponin I (TnI) regulates thin filament activation and muscle contraction, and two isoforms TnI-fast (*TNNI2*) and TnI-slow (*TNNI1*), are predominantly expressed in fast- and slow-twitch myofibers, respectively. Genes encoding these isoforms result in congenital myopathies and/or cardiomyopathies.^120^ Of interest, variants in *TNNI2* (OMIM: 191043) are among the known causes of DA, whereas *TNNI1* was recently implicated in a single family with dominant proximal arthrogryposis with the caveat that pathogenicity of the reported TnI variant (p.K175*) was not further investigated.^121^ Donkervoort *et al*., provided detailed characterization of patients with recessive loss-of-function and dominant gain-of-function variants in *TNNI1*, as the cause of hypo- and hypercontractile muscle phenotypes, respectively.^122^ Functional and contractility studies support that increased Ca^2+^ sensitivity of force resulting in continuous hypercontractility of the muscle fibers is underlying disease mechanism for presumed gain of function *TNNI1* variants in a family with arthrogryposis and elevated serum creatine kinase levels. This variant-specific sarcomeric dysfunction also suggests different targeted therapeutic strategies for recessive and dominant forms of *TNNI1*-related disease.

### Skeletal muscle – intermediate filament

Variants in *FLNC*, the gene encoding filamin C, which is an actin binding protein involved in sarcomere stability and maintenance, are usually associated with cardio- and/or skeletal myopathies, including myofibrillar myopathies and nemaline myopathy. A recurrent *de novo FLNC* variant has been more recently associated with DA with cardiomyopathy.^28,123^

### Other genetic causes of fetal akinesia

The first human phenotype attributable to loss-of-function of the myogenic factor MyoD is perinatally lethal FADS in a single consanguineous family with affected sibships.^124^ The MyoD family of transcriptional regulators is among the earliest described example of a developmental fate-controlling transcription factors and includes two primary-muscle lineage determining factors, *MyoD* and *Myf-5*.^124,125^ To date, there is a handful of patients reported with pathogenic *MYOD1* variants within a spectrum from severe lethal FADS to milder phenotypes. Main clinical features are craniofacial anomalies, respiratory insufficiency, diaphragmatic anomalies, hypotonia, arthrogryposis and renal anomalies. Bilateral vocal cord paralysis in an infant with hand and finger anomalies, inspiratory stridor, hypotonia and respiratory insufficiency is defined as a part of the clinical spectrum,^126^ and phenotype is further expanded with a 38-year-old pregnant woman who presented at 30 weeks’ gestation with respiratory distress and preeclampsia, who had a previous history of slowly-progressive dyspnea, generalized weakness and previous surgery for ptosis and prognathia.^127^

Glycogenosis type IV (GSD IV, OMIM: #232500) or Andersen's disease is a rare autosomal recessive metabolic disorder due to pathogenic variants in *GBE1*, leading to glycogen branching enzyme deficiency and an excessive deposition of structurally abnormal, amylopectin-like glycogen in affected tissues (liver, skeletal muscle, heart, nervous system, etc.). Historically, GSD type IV was classified into six clinical subtypes depending on the hepatic and/or neuromuscular presentation, age of onset and severity.^128,129^ Severe neuromuscular presentations were categorized under fatal perinatal (hydrops fetalis, joint contractures, perinatal death) or congenital/neonatal subtype (severe hypotonia, muscle atrophy, need for mechanical ventilation, and death in the neonatal period or early infancy). Therefore, *GBE1* is included in various diagnostic gene panels such as congenital myopathy, arthrogryposis and fetal akinesia. Recently, a phenotypic characterization score is proposed to evaluate neuromuscular, hepatic and cardiac involvement, thus reflecting the multidimensional clinical continuum.^130^ Lefevre et al., described characteristics of 10 cases from eight families with the severe neuromuscular form of GSD IV in comparison with the literature, including a total of 51 cases (perinatal: n = 16; congenital: n = 35).^131^ Main antenatal features are defined as FADS or arthrogryposis/joint contractures often associated with muscle atrophy, decreased fetal movement, cystic hygroma, and/or hydrops fetalis, whereas presentation at birth was severe with hypotonia and/or muscle atrophy, need for mechanical ventilation, cardiomyopathy, retrognathia and arthrogryposis, resulting in stillbirth or death within the 1^st^ month of life.^132^ The authors highlight the importance of performing a pathological examination of both the fetus and placenta, including unexplained early miscarriages, and considering prenatal exome to facilitate diagnosis.^131^ Of note, out of three cases with the neuromuscular forms of GSD IV in the absence of characteristic polyglucosan bodies on muscle biopsy, which highlights the importance of including *GBE1* in the molecular diagnostic work-up even the absence of hallmark pathology.^132^

### Tissue-specific extracellular matrix (ECM) dysregulation

Recently, Boschann et al., identified biallelic *ADAMTS15* pathogenic variants in five affected individuals from four unrelated families with DA, characterized by congenital flexion contractures of the interphalangeal joints and hypoplastic or absent palmar creases.^133^ Phenotype also includes knee, Achilles tendon, and toe contractures, spinal stiffness, scoliosis, and orthodontic abnormalities. The ADAMTS/L superfamily comprises several metalloproteases and related glycoproteins (ADAMTS-like proteins), which are implicated in microfibril assembly ECM regulating pathways.^131^ Combined with the RNA data, the authors hypothesize that ADAMTS15 plays a critical role in perimuscular connective tissue and tendon development.^134^

## Discussion

Fetal akinesias encompass several etiologies unified by reduced or absent fetal movements; within this review we have focused on Mendelian causes that primarily affect the neuromuscular system (Figure 1). There is an ongoing identification of causative genes, and recognition of multiple inheritance patterns, variable penetrance and expressivity of the known genes, which challenges the evaluation of patients mostly from small pedigrees.

In this genomics era, the boundaries between discrete genetic and clinical entities are becoming increasingly blurred with recognition that variants in a particular gene can frequently give rise to range of phenotypic presentations and disease severities. In 2013, Boycott et al. predicted that many “novel” diseases would represent phenotypic expansions of known disease gene,^135^ more than a third of all OMIM gene entreaties contain multiple phenotype associations.^136^ This is also the case for fetal akinesias, perhaps the most recent and elegant demonstration of this is the expanding phenotypes associated with variants in the genes encoding embryonic myosin heavy chain (*MYH3*), *SCN1A, TOR1A, PI4KA, ARL6IP1*.^17,40–42,112,137–140^ Biallelic pathogenic variants in *PI4KA*, encoding a highly conserved enzyme involved in lipid phosphoinositide metabolism, result in a spectrum varying from autosomal recessive perisylvian syndrome, cerebellar hypoplasia and arthrogryposis to some milder clinical presentations including a variety of immunologic, intestinal, neurodevelopmental and neurologic presentations.^139,140^ Moreover, Parmar *et al*., identified novel compound heterozygous *PI4KA* variants in a family with two affected individuals with a pure HSP phenotype with symptom onset in the teenage years.^141^ As a more recent example, phenotypes associated with biallelic variants in *ARL6IP1,* with a rare, complicated form of progressive HSP, now includes neurogenic AMC with dysmorphic features, ventilator-dependent progressive respiratory failure, and malformations of cortical development.^17^ This adds another layer to the complex cellular pathways involved and shared between AMC and neurodegenerative disorders and highlights that variants linked to later-onset neurodegenerative disorders, can also cause early-onset severe presentations.

It is well recognized that data sharing and international consortia are a key first step to identify the spectrum of rare diseases and causes. Thanks to the International AMC consortium established in 2020 comprising experts in different clinical disciplines (obstetrics, pediatric neurology, genetics, orthopedics, rehabilitation, kinesiology, epidemiology), bioinformatics and affected individuals.^8,9,142^ Among several research pathways, defining common data elements serve as an entry point to multi-institutional implementations and collection of systematic data, which will facilitate new gene discoveries, knowledge translation, and genetic counseling.^143^ The development and implementation of international registries combined with collection of systematic data across various domains from fetal development to adulthood will pave the way for understanding etiology, causative mechanisms, genotype-phenotype correlations and functional outcomes on a more global level.^142,143^

A definitive diagnosis is crucial to guide and tailor management strategies during prenatal and natal periods, and to further provide genetic counseling.^4,8,9^ To date, although there are several recommendations for diagnostic workflow during pregnancy and after birth, there is no uniform consensus.^3,4,14,15,144^

On clinical grounds, a genomic workup for fetal akinesia/hypokinesia of presumed underlying genetic etiology requires teamwork. To navigate the traps for accurate diagnosis of AMC in cases with negative and/or inconclusive exome sequencing, a multidisciplinary team from maternal fetal medicine, radiology, neonatology, pediatric neurology, genetics, and close exchange of clinicians, molecular geneticists, and bioinformaticians play an important role. The yield from clinical genetic testing varies depending on the time of diagnosis (prenatal vs natal period), lethality, presence of other organ and system involvement, CNS involvement, consanguinity, and family history. Comprehensive phenotyping, complementary laboratory and imaging workup, reverse phenotyping, evolution of the clinical findings, and periodic reanalysis can serve as valuable strategies,^145,146^ along with a ‘genomic stepwise check list’ as suggested in other rare diseases’ fields.^146^

Chromosomal microarray analysis (CMA) should be considered in lethal forms, in the presence of dysmorphic features, congenital anomalies, intellectual disability and CNS involvement, since deletions or duplications of chromosomal regions containing key gene/s can also interfere with normal fetal movement. In the case of fetal structural anomalies, the yield from CMA is reported to be 6–10%, in addition to the 14% of chromosomal anomalies detectable by karyotyping.^147–149^ Given the relatively high molecular diagnostic yield for fetal akinesias and the opportunities for an accurate molecular diagnosis to inform genetic counseling and family planning, rapid or timely clinical genomic studies (including comprehensive gene panels, exomes, or genomes) should be considered in all cases of unexplained fetal akinesia and contractures.^26,27,144,148,149^

Mone *et al*., in a cohort of 54 cases with a structural anomaly on prenatal ultrasound, showed that the diagnostic yield before vs. after implementation of prenatal ES was 28% (7/25) vs. 55% (16/29), respectively.^150^ Monitoring of fetal structural anomalies on ultrasound revealed that additional anomalies were detected in 11/15 (73%), and of interest, arthrogryposis in 3/11 (27.3%) of the cases, between the second and third trimesters. Authors also recommend that postnatal follow-up of the evolving phenotype and genomic data reanalysis are critical for maximizing the impact of genomics.^150^ Periodic and systematic reanalysis of genomic data (every 12–18 months) is likely to result in additional new diagnoses.^151^ Automated reanalysis of genomic data will be critical to providing timely molecular diagnoses to families, given the contribution of autosomal recessive disorders to fetal akinesias, this time diagnosis is critical for informing family planning.

For the lethal contractural phenotypes especially combined with postmortem evaluations and deep phenotyping, the yield of ES after a negative array CGH, especially in highly consanguineous populations increase up to 80%.^152^ In this recent cohort including 18 families with 26 affected fetuses, 50% of identified variants and associated phenotypes fell into the subgroup of neuromuscular disorders, with some dual diagnosis requiring complementary further testing, such as Sanger sequencing and fragment analysis, for *CHST14* and *DMPK*, respectively.

Recently, Le Tanno *et al*., based on their experience in 125 patients with AMC with amyoplasia (43%), DA (27%), and other forms (30%), with a definitive etiological diagnosis in 66%, presented a two-step approach: first, to classify patients into above mentioned groups, and second, to shift from invasive procedures with low yield to use of sequencing technologies depending on the subset of patients.^153^

As with other rare diseases, a bottleneck in the definitive molecular diagnosis of fetal akinesias remains with the ever-increasing number of VUSs and the need for functional assays including high throughput approaches and MAVEs.^154^ A relatively low-cost alternative is perhaps ensuring all identified variants within diagnostic and research settings are uploading into gene and variant curation databases such as Clinvar or the LOVD, with increased variant sharing many VUSs may be reclassified, indeed E/Prof Johan den Dunnen suggested that VUS may be an appropriate abbreviation for *Variants of Under Sharing*.^155^ A key to realizing the full clinical diagnostic potential of variant curation is ensuring that sufficient clinical and variant segregation details are also shared within these databases.

Biobanking of patient material including autopsy and/or biopsy material and generating patient-derived cell lines for investigations of the transcriptome and/or proteome can be vital to elucidating the consequence of VUS, in particular cryptic splice-altering variants,^156^ or missense variants that may alter protein stability.^157^ Minigene assays to assess the functional impact of cryptic splice-altering variants can be useful in instances where patient material is not available or where the gene of interest is not expressed in available biospecimens. Validation studies are ongoing to explore how best to implement minigene assays into ACMG guidelines for clinical utility.^158^ In instances where gene expression is low or absent in an available biospecimen (e.g., skin fibroblasts or PBMCs) it is possible to transactivate a gene of interest and then perform cDNA studies or RNA-seq. This is the focus of an Australian Genomics funded project termed PERSYST (Pathogenic Evaluation of Recalcitrant variants by SYStematic Transactivation).^159^

Proteomics is emerging as a powerful diagnostic tool to clarify VUS associated with rare Mendelian diseases.^157^ Given the contribution of loss-of-function variants to neuromuscular fetal akinesias, RNA-seq and proteomics are likely to be informative for many undiagnosed cases. RNA-seq studies and proteomics were recently used to demonstrate that a variant in *SENP7* causes fatal AMC with early respiratory failure and neutropenia.^160^

Platforms to enable sharing of candidate novel disease genes and variants include GeneMatcher^161^ and MatchmakerExchange^162^ continue to empower discovery and diagnosis of Mendelian disease. These platforms are perhaps particularly useful for fetal akinesias which are incredibly rare and extremely genetically heterogenous, and in our experience have been critical to describing novel genetic causes of fetal akinesia.

Given the ever-blurring boundaries between diagnostics and research, and the widespread adoption of exome and genome sequencing as a clinical frontline test, the ACMG have recently published guidelines to aid in streamline of reporting of novel human disease genes.^163^

As with other areas of rare disease genetics, it is likely that some of the missing heritability of fetal akinesias lie within the dark genome,^164^ with long-read genomic technologies including sequencing and optical mapping, new resources including the complete human T2T genome and the pangenome, together with catalogues of structural variants, it is likely that we are entering a new era of genomic discoveries.

It is also likely that some fetal akinesia cases have remained unresolved due to pitfalls in genomic investigations and variant curation, these are perhaps best described in a > 4,500 cohort study by AlAbdi *et al*.^165^

More than 50% of molecularly defined fetal akinesias are due to autosomal recessive or X-linked inheritance, the impact of reproductive or pre-pregnancy carrier screening in this setting cannot be overstated. Several peak bodies including the ACMG,^166^ now recommend that reproductive carrier screening for severe recessive disorders of childhood should be available to all couples, with some countries exploring the implementation of such programs or offering reproductive carrier screening to their populations (e.g., Mackenzie's Mission in Australia.^167^ Reproductive carrier screening, if implemented at population levels, may reduce the incidence and burden of fetal akinesias.^168^

## Conclusion

Precise phenotyping is the first step to facilitate genomic data interpretation. Genetically unsolved AMC cases highlight the importance of multidisciplinary collaborations at every level to identify remaining disease-causing variants, re-classify VUS and navigate the rate-limiting steps of genomic data interpretation and functional validation. Automated or periodic reanalyses are likely to result in new diagnoses, in part due to the number of novel fetal akinesia genes identified. Additional diagnostic pipelines will help to understand the complex regulation of *in utero* movement, and to enable genetic diagnosis and counseling. Monitoring *in utero* movements, fetal anomalies, fetal phenotyping combined with postnatal evaluations will accelerate implementation of prenatal genomic sequencing technologies. Prenatal diagnosis will further aid clinical decision-making, including pregnancy course, delivery, maternal care and neonatal management, and translates invaluable information for alternative reproductive choices and non-invasive prenatal diagnosis. The widespread implementation of preconception carrier screening may see the burden of recessive and X-linked fetal akinesias reduce in those populations.
